# 'I Used to Fight with Them but Now I Have Stopped!': Conflict and Doctor-Nurse-Anaesthetists' Motivation in Maternal and Neonatal Care Provision in a Specialist Referral Hospital

**DOI:** 10.1371/journal.pone.0135129

**Published:** 2015-08-18

**Authors:** Matilda Aberese-Ako, Irene Akua Agyepong, Trudie Gerrits, Han Van Dijk

**Affiliations:** 1 Sociology of Development and Change (SDC) Group, Wageningen University, Wageningen, Netherlands; 2 Ghana Health Service, Accra, Ghana; 3 University of Ghana, School of Public Health, Legon, Ghana; 4 Amsterdam School of Social Science Research, University of Amsterdam, Amsterdam, Netherlands; Örebro University, SWEDEN

## Abstract

**Background and Objectives:**

This paper analyses why and how conflicts occur and their influence on doctors and nurse-anaesthetists' motivation in the provision of maternal and neonatal health care in a specialist hospital.

**Methodology:**

The study used ethnographic methods including participant observation, conversation and in-depth interviews over eleven months in a specialist referral hospital in Ghana. Qualitative analysis software Nvivo 8 was used for coding and analysis of data. Main themes identified in the analysis form the basis for interpreting and reporting study findings.

**Ethics Statement:**

Ethical clearance was obtained from the Ghana Health Service Ethics Review board (approval number GHS-ERC:06/01/12) and from the University of Wageningen. Written consent was obtained from interview participants, while verbal consent was obtained for conversations. To protect the identity of the hospital and research participants pseudonyms are used in the article and the part of Ghana in which the study was conducted is not mentioned.

**Results:**

Individual characteristics, interpersonal and organisational factors contributed to conflicts. Unequal power relations and distrust relations among doctors and nurse-anaesthetists affected how they responded to conflicts. Responses to conflicts including forcing, avoiding, accommodating and compromising contributed to persistent conflicts, which frustrated and demotivated doctors and nurse-anaesthetists. Demotivated workers exhibited poor attitudes in collaborating with co-workers in the provision of maternal and neonatal care, which sometimes led to poor health worker response to client care, consequently compromising the hospital's goal of providing quality health care to clients.

**Conclusion:**

To improve health care delivery in health facilities in Ghana, health managers and supervisors need to identify conflicts as an important phenomenon that should be addressed whenever they occur. Effective mechanisms including training managers and health workers on conflict management should be put in place. Additionally promoting communication and interaction among health workers can foster team spirit. Also resolving conflicts using the collaborating response may help to create a conducive work environment that will promote healthy work relations, which can facilitate the delivery of quality maternal and neonatal health care. However, such an approach requires that unequal power relations, which is a root cause of the conflicts is addressed.

## Introduction and Analytical Concepts

Health workers’ motivation can be defined as individuals’ *‘degree of willingness to exert and maintain an effort towards organizational goals’* [1]. Motivation is affected by internal psychological as well as transactional processes and interactions between individuals and their work environment as well as the broader societal context [1]. As such, individual workers’ motivation can be affected by conflicts between collaborating workers. Individual, interpersonal and organisational factors contribute to conflicts and unequal power relations and relations of distrust among workers can affect how workers respond to conflicts [2–5]. Poor responses to conflicts can lead to persistent conflicts, which can frustrate efforts towards achieving organisational goals. Conflicts can be particularly devastating where care provision is dependent on cooperation, understanding and interdependence between different professional groups.

Health worker motivation has been widely studied using a variety of qualitative [6–8] and quantitative methods [8,9]. In Ghana, low motivation of health workers in maternal and neonatal health care provision has been cited as one of the factors impeding the achievement of the set targets of Millennium Development Goals 4 [Reduce child mortality] and 5 [Improve maternal health] by 2015 [10–14].

There is however very little published literature examining inter professional conflicts and their role on workers’ motivation, care provision and outcomes for maternal and newborn health in low and middle income countries [3–5]. This paper therefore aims to contribute to knowledge in this area by presenting the findings of an indepth exploration of why and how unequal power relations and relations of distrust can lead to conflicts, which in turn affect the motivation of doctors and nurse-anaesthetists in the provision of maternal and neonatal health care in a specialist public hospital in Ghana, a lower middle income sub-Saharan African country. Understanding the links between conflicts, power, trust/distrust and motivation in health care organisations can contribute towards finding solutions to improve health workers’ motivation and performance, which can help accelerate progress towards achieving the unfinished Millennium Development Goals 4 and 5 agenda [7,10–13,15,16].

We theorized that health workers’ motivation can be influenced by conflicts resulting from personal, interpersonal and organisational factors. How individuals and organisations respond to conflicts is influenced by trust and power relations. Where there is distrust, disagreements can be interpreted with suspicion, which can influence responses that can intensify conflicts. Also where there is contestation of power, workers’ desire to assert themselves can intensify conflicts. When appropriate responses are not employed to resolve conflicts, workers may lose trust in co-workers and the organisation, become less motivated and exhibit poor attitudes in collaborating with co-workers and a cycle of conflicts in worker relations develops. Persistent conflicts may hinder the achievement of organisational goals. We therefore draw upon the concepts of conflict, trust and power relations to guide our analysis.

### Conflict

Conflict can be defined as a difference in opinion [17]. Most authors agree that conflicts in health care organizations result from individual differences, interpersonal differences and organisational arrangements and gaps [2,18–20]. Classical organisational theory and the human relations school suggest that conflicts have negative consequences on organisations, because conflicts prevent organisations from achieving their goals [21]. From this perspective, conflicts should be avoided. However other studies suggest that organisational conflicts should not be necessarily perceived as negative [2,17,22]. Rather the way the parties to a conflict perceive and respond to it determines its potential negative or positive consequences on an organisation. Conflicts can have positive consequences if they encourage self-examination, lead to healthier relations, enhance workers’ motivation and contribute to organisational transformation [21,23,24]. Zydziunaite and Katiliute [25] found that when conflicts between co-workers are resolved constructively, their motivation increases, which impacts positively on their performance. However even this school of thought also agrees that persistent conflicts if not constructively resolved can be dysfunctional, negatively affecting psychological and physical wellbeing, motivation, job satisfaction and performance of workers [5,24,26,27].

Three major causes of conflicts in health care organisations have been proposed [2,20,22] as follows: 1) Individual characteristics such as differences in values and demographic dissimilarity; 2) Interpersonal factors among co-workers such as lack of trust, injustice or disrespect and inadequate or poor communication; and 3) Organizational factors including interdependence between different professionals, organisational changes due to restructuring, and asymmetric power relations in health care institutions.

Whetten et al. [21] categorise how individuals and organisations manage conflicts into five possible responses including forcing, accommodating, avoiding, compromising and collaborating. ‘Forcing’ refers to situations where a party attempts to satisfy his/her need at the expense of the other. Strategies employed include the use of formal authority, physical threats, manipulation ploys or ignoring the claims of the other. ‘Accommodating’ refers to situations in which a party tries to satisfy the concerns of the other at the expense of his/her own. ‘Avoiding’ occurs when the interests of both parties are neglected by side-stepping the conflict or postponing the solution. A ‘compromising’ response occurs where each party gives up something to resolve the conflict. It can cause a rapid resolution of conflict but may not resolve underlying problems. ‘Collaborating’ or ‘cooperating’, which is also referred to as the ‘problem-solving’ response, tries to find solutions to the cause of the conflict that are satisfactory to both parties. It takes time and expertise to resolve conflicts by collaboration, and not all conflicts are amenable to being solved that way. Whetten et al. [21] suggest that when the time and expertise is available to use it collaborating can be the most effective approach, since in the end, both parties perceive that their interests have been met. So resolutions tend to be long lasting.

### Trust

Trust especially in hospitals with multi-disciplinary, specialized and interdependent professionals is considered to be crucial to employee commitment and performance [28–31]. Co-worker trust is ‘*confidence that one’s colleagues are competent and will act in a fair*, *reliable and ethical manner*’ [32]. This implies a two-way relationship between the ‘trustor’, defined as ‘*the person who trusts*’ [33] and the ‘trustee’ defined as ‘*the person (s) trusted by the trustor*’ [33]. Relations of distrust among co-workers in a hospital can contribute to conflicts, which can impact negatively on interpersonal interactions, motivation and performance [32,34–38]. The contrary is true. Gilson et al. [30] found that trust among health workers is a motivator to workers’ performance and fosters a conductive work climate.

### Power

Mintzberg [39] describes power in organisations as ‘*the capacity to effect (or affect) organisational outcomes*.’ Various groups or actors within the organisation have different levels of power. In a medical situation, the frontline health workers—such as nurses, anaesthetists and doctors, who Mintzberg refers to as ‘operators’ are highly skilled and knowledgeable and are among the most powerful actors. Their expertise gives them discretionary power [37,39] and they carry out the actions, which are the basic outputs of the organisation. Lipsky [40] labels this group of actors as ‘street-level bureaucrats’ and suggests that their decisions and actions in implementation effectively become public policies. Health workers can influence decisions by exercising power through the use of threats, sabotage, etcetera. This can result in and intensify conflicts [39,41].

## Methods

### Study setting

The hospital (alias Adom hospital) in which the study was conducted, has several specialist departments, which provide general as well as specialist outpatient and inpatient care and walk-in services. In addition, it serves as a referral hospital for smaller surrounding public and private health care facilities. It has a surgical theatre and a maternity theatre equipped to provide the full range of emergency obstetrics care. At the time of data collection, the central management comprised the medical director, accountant, administrator, and the directors of nursing and clinical services. Each specialised department was semi-autonomous and had a departmental head who was accountable to the medical director.

The study was conducted in the obstetrics and gynaecology (O and G) department, which had two administrative sub-units: a nursing unit and a medical officers’ unit. Anaesthesia, was also an independent department and was headed by a senior nurse-anaesthetist. The department, served the anaesthesia needs of the surgical, O and G and eye departments.

### Study design

The first author, MA, also referred to as ‘the researcher’ conducted hospital ethnography. She spent a total of eleven months as a participant observer in the hospital as part of her PhD thesis research. The extensive engagement was to enable her to observe, experience, understand and represent the lives of subjects in their natural setting [42,43]. In the first nine months, MA was an active observer and participant in the process of health care provision in the O and G department; conducting tasks such as assisting nurses to deliver babies, watching over clients in the wards, while nurses followed up on laboratory test results etcetera. Additionally, she collected data through conversations and in-depth interviews. After preliminary analysis of the data, she returned to the hospital for two more months to clarify gaps in the data by conducting further interviews and conversations. Table 1 provides a summary of the number of interviews and conversations by staff category.

**Table 1:** Categories of workers included in conversations and interviews.

Categories of workers	Data collection methods	
Category of workers	Conversations	Interviews
Nurses and midwives	62	12
House officers	5	2
Doctors	11	4
Nurse-anaesthetists	5	3
Ward Aids	2	2
Orderlies	6	6
Doctors who left the hospital	-	2
Laboratory officials	-	2
Department managers	9	1
Hospital managers	3	4

Ethical clearance was obtained from the Ghana Health Service Ethics Review board (approval number GHS-ERC:06/01/12) and from the University of Wageningen. Entry to the hospital was gained through an introduction from the regional director of health services. Within the hospital, one of the senior hospital managers introduced the researcher to the O and G department staff as a student researcher. They were informed of the objectives of the study, and they gave her permission to participate in and observe activities in the wards, at meetings and training programmes. Written informed consent was obtained from all interview participants. Verbal consent was obtained for conversations. To protect the identity of the hospital and research participants pseudonyms are used throughout this article and the part of Ghana in which this study was conducted is not mentioned.

### Analysis

Notes from observations and conversations were refined and expanded at the end of each day in line with standards in ethnographic studies [43]. Interviews were recorded and later transcribed verbatim by a research assistant. The data were typed, grouped and saved in two data sets: 1) interviews 2) observations and conversations. They were then analysed in Nvivo [version 8] by systematically coding to identify common patterns, differences and contradictions. Common patterns from both sets were then grouped into five major themes: incidences of conflict, causes of conflicts, category of professionals engaged in conflicts, how conflicts were managed, effects on health workers and quality client care.

In the analysis, any situation of disagreement between two health workers scheduled to collaborate in the provision of health care was labelled as conflict. Causes of conflicts were grouped into personal, interpersonal and organisational. How conflicts were addressed were categorised into five sub themes in line with Whetten et al.'s [21] five conflict management responses.

When workers expressed sentiments including feeling emotionally low, lack of job satisfaction etcetera, as emanating from conflict experiences, this was labelled as negative effects of conflicts on health workers’ motivation. Situations where conflicts contributed to delay and/or failure to provide care were labelled as negative effects of conflicts on quality client care.

Positive consequences of conflicts on health workers’ motivation included: 1) situations where workers expressed personal satisfaction with the way conflicts were resolved 2) when an incident of conflict resulted in standard procedures being followed in the provision of health care.

The majority of conflicts were between doctors and nurse-anaesthetists. This appeared to be the main area where conflicts were affecting motivation and performance. The rest of this paper therefore focuses on these conflicts.

## Results

While accompanying a midwife on her ward rounds, MA begun to converse with her about challenges to maternal and neonatal health care provision in Adom hospital. The midwife said:

They [nurse-anaesthetists] are the cause of most of the problems in the theatre. They decide when they want to come, when they want to do a case and which case they will want to do. They sometimes decide that they will not do elective cases, but will only do emergency cases.

**Fig 1:**
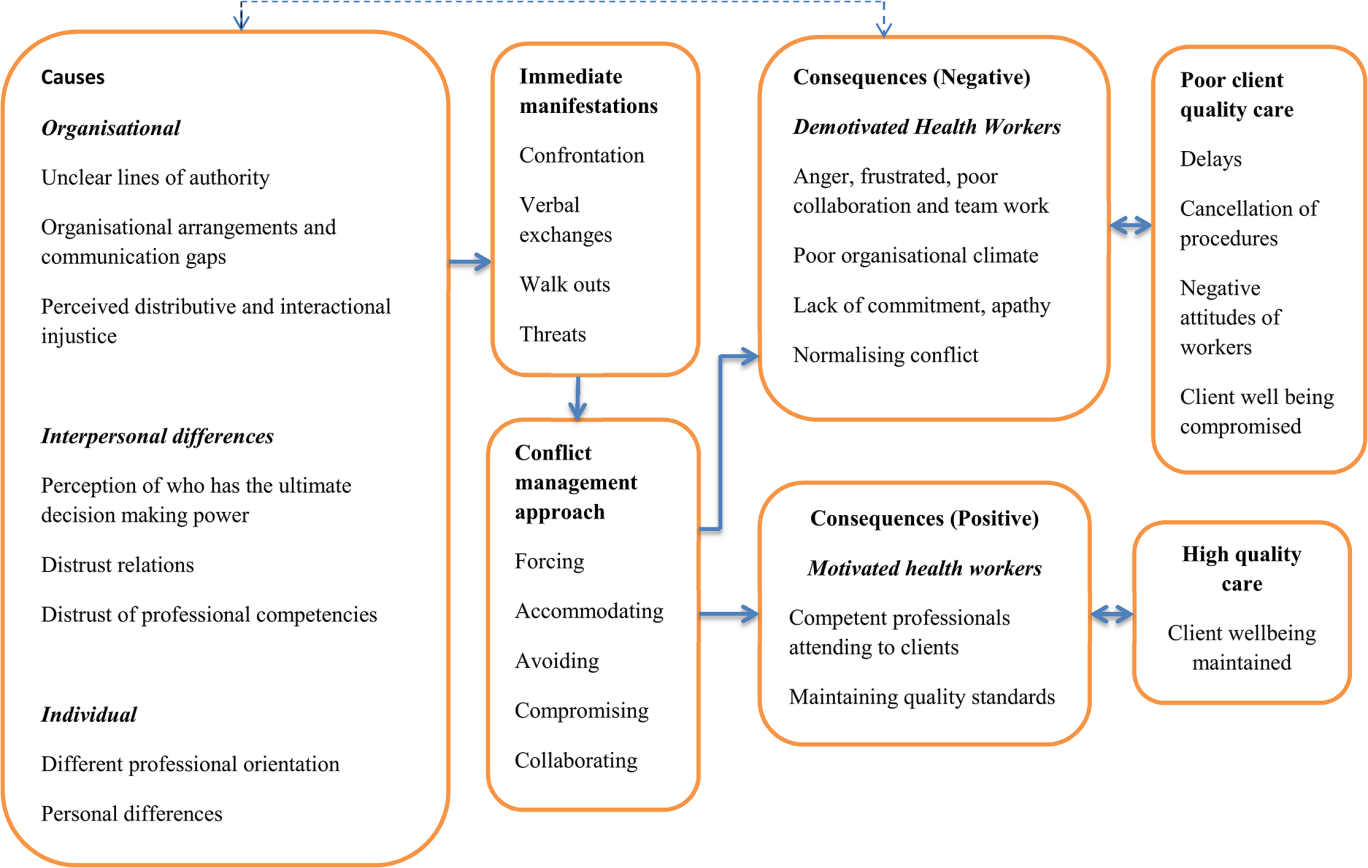
Framework of causes, management and consequences of conflicts in Adom hospital. A summary of the study results indicating the following: the three main causes of conflicts; the immediate manifestations of conflicts; Whetten et al.’s (1996) five conflict management responses adopted in the study; the negative consequences of conflicts on health workers’ motivation; the negative effects of low health worker motivation on poor quality client care; the positive effects of conflicts on worker motivation and also the effects of high worker motivation on quality client care.

This was one of the many occasions that the researcher heard or observed in meetings, conversations and workshops, complaints about nurse-anaesthetists. So she decided to devote some time in the operating theatre, to observe the interaction between doctors and nurse-anaesthetists. An incident observed on a morning when the theatre was bustling with activities is illustrative of the several incidences of conflicts that MA observed between doctors and nurse-anaesthetists in the hospital. The causes of the various conflicts appeared to be similar and included individual, interpersonal and organizational factors, with deeper roots of power struggle and distrust relations between doctors and nurse-anesthetists (see summary in Fig 1). We first present this conflict and then disentangle the causes of this and other conflicts the researcher observed, and the way they were addressed (using the five responses as proposed by Whetten et al. [21]). In addition, we illustrate how these conflicts and the way they were addressed affected health workers’ motivation.

Dr. Hilary, a senior doctor, Dr. Kofi, a junior doctor and Dr. Kumoji, a house officer were on duty in the O and G department. Dr. Hilary stayed in the consulting room, while the other two worked in the operating theatre. Mr. John, a senior nurse-anaesthetist and Ms. Joan, a junior nurse-anaesthetist were scheduled to work with the O and G and the surgery departments respectively. The first emergency obstetric case for the morning was a pregnant woman with a diagnosis of placenta previa type 2, who was bleeding profusely. Fortunately there was still a foetal heartbeat, and an emergency caesarean section (C/S) to save mother and neonate was arranged. The mother was wheeled into the operating theatre where Dr. Kofi and Dr. Kumoji were waiting for Mr. John, to anaesthetise her. Mr. John turned to Dr. Kofi and asked: ‘*Have they done blood count*?’ [Blood count tests offers information that the doctor can use to determine the client’s health status [44]. In this hospital it is the responsibility of the doctor to request the test and instruct the nurses to liaise with the laboratory to conduct the test before surgery]. Dr. Kofi retorted: ‘*Don’t ask me*!’ Mr. John calmly repeated his question: ‘*I am asking whether they have done the blood count*?’ Dr. Kofi retorted: ‘*You have had this coming since morning*. *You should have asked this question before the patient came in*. *I am not the one to answer that question*!’ To which Mr. John now seemingly irritated replied: ‘*Then I will leave the patient*!’. ‘*Yes*, *you can leave*, *after all what is it*! *I will also leave*!’ Dr. Kofi retorted angrily. Mr. John also visibly angry ordered his assistants: ‘*Wheel the patient out*, *we won’t do it*!’

Dr. Kofi and Mr. John continued the angry exchanges as both men stormed out of the theatre. Some of the nurses, orderlies and anaesthetic assistants present appealed to them to stop arguing, because the patient was suffering, but both men ignored the pleas. Then the theatre in-charge said to the pregnant woman who was sitting on the theatre table and moaning: ‘*You too*, *they are fighting because of you and you are here crying*!’ About ten minutes after their disappearance, Dr. Hilary arbitrated in the incident using a ‘compromising’ approach [22]. She asked Dr. Kofi to return to the theatre and Ms. Joan, the nurse-anaesthetist originally assigned to the surgical theatre, was called in to give spinal anaesthesia. Dr. Kofi safely delivered the mother of a live baby within thirty minutes.

Subsequently we explore the causes, responses and consequences of this conflict as well as other conflicts between doctors and nurse-anaesthetists in the rest of the results section.

### Unclear lines of authority at organisational level and perceived lack of recognition of nurse-anaesthetists

An organisational factor of unclear lines of authority between doctors and nurse-anaesthetists, coupled with an interpersonal factor of perceived lack of recognition of nurse-anaesthetists, contributed to the occurrence of conflicts.

Several informants suggested that there was no clear definition between the doctors and nurse-anaesthetists as to which of the two professional groups had the ultimate authority to decide whether a client should undergo surgery. Both doctors and nurse-anaesthetists felt that the discretionary nature of their work, gave them this authority. The nurse-anaesthetists, felt that the doctors were wrongly assuming that they should take instructions from them because they are originally nurses. A nurse-anaesthetist explained: ‘…*because here we are all nurse-anaesthetists*, *they [doctors] think we have no right to suggest to them to cancel a case*, *so they get upset about it*.

A house officer in an interview suggested that while it was easy to get nurses to take instructions from doctors, it was nearly impossible to get nurse-anaesthetists to take instructions from doctors. A senior doctor also stated in an interview: ‘*They [nurse-anaesthetists]*… *have some airs around them that without them a case cannot be done*.*… So initially I used to fight with them*, *but now I have stopped*.*’*

The majority of the nurse-anaesthetists said that their motivation for pursuing the anaesthesia profession was to gain independence from doctors and to become more empowered. They added that unlike nurses who take instructions from doctors, nurse-anaesthetists take their own independent decisions on which cases to attend to. They suggested that being nurse-anaesthetists boosted their morale and made them feel important. So they resisted collaborating with doctors on occasions that they perceived the latter were trying to impose their authority on them.

Follow up conversations with the different parties connected to the described incident, revealed that Mr. John had decided on his own discretion to work on an elective hernia case that morning with a surgeon from the surgery department, even though he was scheduled to work with the O and G department. Dr. Kofi said he had earlier sent a nurse to inform Mr. John of the impending emergency C/S. However Mr. John had asked him to wait for the hernia case to be done first or refer the C/S case to the polyclinic. Dr. Kofi said he disagreed with Mr. John’s request, because Adom hospital was the main referral hospital within that geographical area that was most competent to perform the emergency C/S. Also in his opinion, it was too risky to refer a bleeding woman to another facility, as it would delay emergency care provision, which could result in the death of mother and child. The surgeon who worked on the hernia case with Mr. John, said the information about the emergency case was not communicated to him, else he would have postponed his case, which was not an emergency.

Similar incidences of conflicts in decision-making on client care between doctors and nurse-anaesthetists were observed on several other occasions. Senior doctors asserted authority over nurse-anaesthetists by resorting to ‘forcing’. For example, a senior doctor stated:

Once, I had a case of ovarian cyst and when I took her to the theatre, there was a female nurse-anaesthetist who asked me ‘Have you done HIV/AIDS test? If you haven’t done the test on the patient, I won’t administer anaesthesia.’ I told her ‘It is not your business to ask whether a patient has been tested for HIV/AIDS or not.’

On the other hand, some junior doctors used ‘accommodating’ response by agreeing to the nurse-anaesthetists’ decisions. For instance a junior doctor, had completed a C/S case and was preparing to perform the next C/S, when the nurse-anaesthetist on duty asked him to suspend all elective C/S and perform only emergency C/S, because he wanted to go somewhere. The junior doctor insisted to know where the nurse-anaesthetist wanted to go, however, the nurse-anaesthetist refused to disclose it. When the nurse-anaesthetists left the scene, the doctor cited the attitude of the nurse-anaesthetist as one of the issues that demotivated him from working in the hospital (Observation notes, 12/02/2012).

Thus nurse-anaesthetists exercised power by using ‘avoiding’ response such as coming to work late and closing early. Sometimes they suggested that they were tired and other times they chose to work with particular doctors that they felt comfortable with. While senior doctors sometimes resorted to ‘forcing’ junior doctors either used accommodating response or reported the poor conduct of nurse-anaesthetists to the senior doctor responsible for the O & G department. The senior doctor in turn either reported such cases to the head of anaesthesia or the hospital director. The director in turn referred some of the cases back to the head of anaesthesia for action to be taken. However observations and discussions with the head of anaesthesia suggested that he only issued verbal reprimand to the culprits.

Persistent conflicts frustrated both doctors and nurse-anaesthetists and demotivated them from collaborating, which occasionally delayed health care provision.

### Different professional orientation resulting in different decision making pathways

Different professional orientation of doctors and nurse-anaesthetists, contributed to different views and decision pathways on client care, which sometimes contributed to conflicts.

Nurse-anaesthetists suggested that compared to doctors they had a greater responsibility to determine if clients were fit for surgery and its aftermath. Additionally from the nurse-anaesthetists’ point of view, not all clients proposed for surgery needed it. Also they perceived that if a client died, they would be held responsible and that most of the doctors were not interested in the welfare of clients, but were interested in just conducting surgeries. So the nurse-anaesthetists became upset when clients were brought into the theatre for surgery without undergoing the necessary laboratory examination and made statements like:

The doctors are supposed to request and ensure that the various lab tests are done, the necessary preparations are made, but they won’t follow up. So cases are brought (to the operating theatre) with a lot of things undone.

The doctors will recommend the patient for surgery, but will not bother to ensure that the necessary preparations [routine laboratory tests] are done. … you will see the client being brought to the theatre and … in the client folder, it is clearly stapled neatly ‘lab test required’ and there is nothing done. It can be so annoying and frustrating. … definitely you will compromise with quality and punish the client.

A hospital management team member supported the nurse-anaesthetists’ point of view. He suggested that doctors sought to satisfy their interest at the expense of the nurse-anesthetists’ efforts:

If a doctor finishes an operation and the patient is not well, it becomes the nurse-anaesthetist’s headache. He has to keep the patient alive, resuscitate the patient. … So that is why they don’t agree with the doctors.

On the other hand, the doctors felt that they were the ones who knew what was best for clients and had the greatest responsibility in client care. They needed to make quick decisions on client care in order to avoid delays, which could result in a client’s death, since they would be blamed for any mishaps.

… these delays, and by the time you are ready to do the surgery the patient has bled so much. Now when you go in to do the surgery DIC (Disseminated intravascular coagulation) could occur, then it will now be the doctor’s responsibility to … save the patient’s life. At that critical point it is now left with you the doctor to try to arrest any situation that could cause the death of the client, because when the client dies, you will be blamed.

The doctors perceived that nurse-anaesthetists and other collaborating workers did not share their view on client care, which they suggested contributed to delays and conflicts in decisions on client care. The doctors also felt that a team must have a leader and they were the natural leaders, as a junior doctor stated in an interview:

Sometimes they [nurse-anaesthetists] think about the complications for you [doctor] and want to decide that this is not a case that should be done this way. But I mean invariably there is supposed to be a leader of the team.

During a maternal audit meeting, a senior nurse appealed for clear guidelines on client diagnoses for C/S, to help reduce such doctor-nurse-anaesthetist conflicts. In response, a doctor said that the nurse-anaesthetists were the cause of the conflicts in the theatre, because they refused to comply with doctors’ decisions.

Consequently doctors and nurse-anaesthetists’ different professional work orientation contributed to conflicts in perception of urgency of client care, which influenced each group’s response to client care. The two groups responded to conflicts by using ‘forcing’, ‘avoiding’ and sometimes ‘accommodating’ response. Most of the conflicts were not resolved, so they persisted.

The repeated conflicts contributed to feelings of anger and frustration. Consequently a poor organisational climate emerged and it demotivated both professional groups from collaborating in the provision of health care.

### Distrust of co-workers’ professional competencies

An interpersonal factor that created conflicts that impacted on the motivation of doctors and nurse-anaesthetists were attempts to prevent workers whose competencies were distrusted from performing procedures.

Due to the scarcity of doctors in the O and G department, sometimes junior doctors and house officers performed tasks that some senior theatre nurses and nurse-anaesthetists deemed beyond their level of competence. Nurse-anaesthetists indicated that they did not feel comfortable when junior doctors and house officers who were supposed to work under the supervision of senior doctors, were allowed to perform complicated surgery cases unsupervised. Nurse-anaesthetists explained that in order to protect clients from poor quality care, they resisted such attempts by recommending that more senior persons performed the surgeries, or gave excuses that the client was not ready for surgery or openly refused to collaborate. However the junior doctors got upset as a nurse-anaesthetist narrated:

We prefer that the senior doctor does it [emergency C/S]. So that you do not risk the life of the client. And the complaints we hear is that we are trying to frustrate the young doctors. Their [the doctors] head will come telling us that ‘*eh* my young doctors want to learn, but you are trying to frustrate them.’ How can you use human beings as guinea pigs! So we tell them: ‘Well if you want to do so, then we do not want to be part of it.’

Similarly, doctors who did not trust the competencies of nurse-anaesthetists that they were scheduled to work with preferred to invite a senior anaesthetist to perform the anaesthetic procedure. A senior doctor shared an experience:

… there was a certain patient that I operated on and that patient was not producing urine and I thought that this nurse-anaesthetist who was on duty should have called the doctor-anaesthetist [a senior anaesthetists, who was on study leave at the time of the study]. When I mentioned it, she nearly had some verbal exchange with me and so I kept quiet…We did the case and after the case, the patient was not passing urine … So I had to call the doctor-anaesthetist to come and review the case.

So doctors and nurse-anaesthetists used ‘collaborating’, ‘avoiding’ and ‘forcing’ responses to prevent colleagues whose competencies they distrusted from performing procedures.

Both nurse-anaesthetists and doctors who succeeded in ensuring that distrusted colleagues did not perform procedures, said they derived inner satisfaction from occasions that they were able to ensure that experienced persons performed procedures. For instance the senior doctor expressed his satisfaction that the doctor-anaesthetist, who is more experienced, came in to save the client. Also, the nurse-anaesthetists said they were always happy whenever they succeeded in preventing a doctor wo was deemed incompetent from performing a procedure on a client. On such occasions clients received quality care, so such workers felt motivated.

### Locum practice and illegal charges and the inability of the hospital to resolve such matters

Most of the private health facilities within Adom hospital’s catchment area engaged the services of some of the skilled and experienced doctors and nurse-anaesthetists in Adom hospital as locum (private practice) staff. This sometimes resulted in scheduled doctors and nurse-anaesthetist reporting late or absenting themselves from work. Such practices contributed to delays, cancellation of procedures and sometimes emergency cases had to be referred to other facilities. Aggrieved workers repeatedly reported such practices to hospital managers, but managers did not seem to be able to get the culprits to desist from such practices. So some workers became frustrated, lost faith in management and resigned themselves to sharing such workers with private facilities–even when such workers were scheduled to be on duty in the hospital. A senior doctor shared an experience:

Whenever he [nurse-anaesthetists] was on duty you had to virtually share him with several other facilities, for he was working in several other facilities. So you will be working with him and he will say he is coming, then he will disappear for up till two hours.

A nurse-anaesthetist admitted that most doctors and nurse-anaesthetists were doing locum, but he distrusted doctors who sacrificed their schedule in Adom hospital to do locum. He cited an instance when a client was sent from the study hospital to a private facility, because the scheduled doctor was absent. It turned out that the absent doctor who was scheduled for duty in the study hospital was the very doctor attending to clients in the private facility

So when a scheduled doctor or a nurse-anaesthetist delayed or was absent from work it was suspected that the worker was engaged in locum. A nurse-anaesthetist described the state of suspicion and its consequent influence on workers’ motivation:

People just go about doing whatever they feel like doing and earning their salary, but there is no joy and there is too much suspicion. Now when a doctor or a nurse- anaesthetist is not there, the perception is that the person is gone to do locum.

Additionally nurse-anaesthetists and nurses suspected that some doctors brought in clients from their locum practice and secretly charged them illegal fees. So they felt it was unfair to collaborate with such doctors on such cases, because such clients were not their responsibility. A nurse-anaesthetist shared her suspicion:

… there were three emergencies … the senior person [doctor] convinced us that he could do all three cases within a stretch of two hours, so if we could sacrifice to stay, we could work on the cases. …Then he brought up a myomectomy case [refers to the surgical removal of uterine leiomyomas [45]] and convinced us that he will work on it first and then he will quickly work on the emergencies. Being a specialist we all rationalized that …with his skill and level of expertise he will be able to finish the myomectomy, which wasn’t any of the emergencies and quickly work on the other three cases and we will be done in no time, so we agreed. Now he finished the myomectomy case and he called a very junior doctor to come and do the emergencies and left.

The nurse-anaesthetist further stated:

I have lost trust in him [senior doctor]. For all you know he had an arrangement with the myomectomy client. By now he told her ‘You come and I will do this for you for this amount.’ So he had taken his money from the client and we had to work for it.

Distrust relations and perception of unfairness in rewards from illegal charges were also noted in connection with the incident in the theatre, described earlier. In a follow up conversation with Mr. John he confirmed his suspicion that Dr. Kofi brought in the pregnant woman from his locum practice. He asserted that if the woman had come through one of the wards in the hospital routine laboratory tests including a blood count would have been done. He suspected that Dr. Kofi was benefiting alone from an illegal deal with the client. He said the doctor’s illegal activities had been reported to the hospital management, but hospital management was reluctant to discipline him, because the O and G department had few doctors, so they could not risk losing him.

Conversations and interviews were conducted with department and hospital managers to understand how illegal charges were addressed at organisational level. A nurse manager explained that it was illegal for doctors to charge clients fees, and the hospital’s accounts department was responsible for such matters. Most obstetrics and gynaecological services were free, as per the fee free delivery service policy of the government. She added that as a nurse manager she had no power to discipline doctors, so she reported doctors who charged illegal fees to the head of the doctors, who had the power to discipline them. She cited an instance that she reported a house officer who repeatedly charged clients illegal fees to the senior doctor acting as the head of the department and the hospital administrator. The senior doctor reprimanded him, but she had no idea how the administrator addressed the case.

Interviews with two hospital management members revealed that workers were free to practice locum, once it did not affect their work schedule. Nevertheless one said that it was nearly impossible to get evidence of workers who shuttled between their jobs and locum practice, because their colleagues shielded them. The other manager, who was the head of the hospital, however admitted that she had had repeated confrontations with one of the senior doctors in the O & G department over his frequent locum practice, at the expense of his work in the hospital. Both managers also admitted that illegal charges existed. The other who was the hospital administrator, said she only heard about them, but she had no evidence suggesting that workers engaged in it. In the case of the house officer who charged illegal fees, she said she had been informed, but she had no power to discipline doctors, only the head of the hospital had such power. The head explained that illegal charges were not reported when collaborating parties shared the money, but conflicts arose when the benefits were not distributed among collaborating parties. She cited the case of a nurse-anaesthetist who reported the illegal deals of a doctor to the hospital management. When it was investigated, it was realised that the doctor shared the benefits with her colleagues, which she was not privy to. She added that workers were free to report illegal practices to their immediate superiors or to hospital management for the necessary disciplinary actions to be taken.

The lack of a clearly defined organisational reporting system and strategy to deal with illegal charges and locum practice contributed to distrust relations between doctors and nurse-anaesthetists. Both doctors and nurse-anaesthetists who reported suspicious cases of locum and illegal charges, felt that the department and hospital managers did not adequately address their concerns. Consequently they took matters into their own hands by using ‘avoiding’ and ‘forcing’ responses to deal with such cases, which rather intensified conflicts and sometimes delayed health care provision.

### Organisational arrangements inhibited frequent communication and the building of team spirit

The organisational work arrangements and practices limited interaction and created gaps in communication between collaborating workers, which contributed to conflicts. Firstly a doctor on duty conducted daily ward rounds and on Monday mornings a senior doctor led a team of doctors to conduct general ward rounds. Such rounds were used to review, discuss and decide on surgery cases. However nurse-anaesthetists were not privy to such decisions, because they did not participate in such rounds. Secondly, being a specialist referral hospital, it received emergency obstetrics and gynaecological cases referred from other facilities. Doctors sometimes sent such emergency cases directly to the theatre, without involving the nurse-anaesthetists. So doctors and nurse-anaesthetists mostly met and interacted in the midst of surgical procedures. A management member described the situation:

Normally they [nurse-anaesthetists] don’t see patients. Surgeons and gynaecologists see all the patients. They only see the patients when they are going to sleep [being anaesthetised] and after that they only see them briefly and that is all.

The limited interaction between doctors and nurse-anaesthetists as well as the latter’s non-participation in initial decisions on surgery cases, heightened their suspicion that some of the clients were not emergency referrals, but were individual doctors’ private clients. So nurse-anaesthetists collaborated well with particular doctors they trusted, while they sometimes avoided collaborating with doctors they distrusted. For instance on one occasion a team of doctors had to delay an emergency C/S referral case that was brought the previous day. When the doctors were ready to perform the procedure, the scheduled nurse-anaesthetists and other theatre workers closed at 1.30pm, instead of the official 2.00pm. So the doctors had to wait for the staff scheduled for the afternoon shift to arrive and settle in, before the emergency C/S could be performed (Observation notes, 23/07/2012). A senior doctor lamented over other professionals’ poor attitude towards collaborating:: ‘*Sometimes when you want to do a C/S*, *you have to convince the nurse-anaesthetists*, *convince several people*.’ He added that he started to work in the hospital with enthusiasm, followed by persistent conflicts to get co-workers to collaborate. Subsequently he became frustrated, apathetic and demotivated. He said one day after quarrelling with co-workers to get work done, his blood pressure shot up, so he stopped quarrelling with them and ‘had become like them’ [exhibiting lackadaisical attitude to work], because he needed to stay alive. Also a junior doctor expressed his frustration with the culture of collaborating: ‘*…sometimes there is a lot of resistance* [from nurses and nurse-anaesthetists]. *You have to be very very friendly*, *so that you are best friends with them before things are done*.*’*

Nurse-anaesthetists used ‘avoiding’ responses such as leaving work early or they were tired, so they could not work on a case, to deal with situations that they perceived the client to be the doctor’s client. Sometimes doctors had no option but to resign themselves to ‘accommodating’ response towards nurse-anaesthetists who were reluctant to collaborate with them.

Consequently organisational arrangements and communication gaps hindered effective interaction between doctors and nurse-anaesthetists, which contributed to a lack of team spirit. It further bred suspicion and distrust relations that affected decisions on client care, which sometimes led to conflicts.

### Distrust resulting from perceived distributive and interactional injustice

Interpersonal factors including perceived distributive and interactional injustice contributed to distrust relations between doctors and nurse-anaesthetists, resulting in conflicts and worker demotivation.

In particular, the nurse-anaesthetists perceived distributive injustice in their treatment by the hospital management. Nurse-anaesthetists suggested that the hospital gave doctors incentives including fuel coupons and phone-call credits among others, but other workers were not given such incentives.

Also nurse-anaesthetists perceived interactional injustice in their claim that other professionals perceived them as disrespectful and uncompromising, but they felt that was untrue. Additionally they perceived interactional injustice by claiming that other workers raised concerns when nurse-anaesthetists failed to report to work, while nobody raised concerns when doctors failed to report to work. The head of anaesthesia illustrated this perception in the following narration. On one Sunday evening no surgeries could be performed in the O & G theatre, because it was reported that the nurse-anaesthetist scheduled for work was absent. The O & G department management team convened a meeting with the head of the anaesthesia department to address this problem and other complaints that scheduled nurse-anaesthetists came late or did not come to work. The conflict was resolved using ‘forcing’ strategy. The O & G department management blamed nurse-anaesthetists for perceived negative attitudes and also blamed their head for not disciplining nurse-anaesthetists who misbehaved. It was reported that the head admitted the shortfall and promised to convene a meeting with the other nurse-anaesthetists to address the issues. Relations between nurses, nurse-anaesthetists and doctors improved for about three months before the complaints about nurse-anaesthetists resumed. So the researcher conversed with the head of anaesthesia to understand how he addressed the issues from the meeting. He explained that the allegation levelled against the scheduled nurse-anaesthetist was untrue, as the nurse-anaesthetists later explained to him that he had briefly stepped out of the theatre, but the O & G department concluded that he had vacated post. The head therefore suggested that the other professionals sometimes made false allegations against them.

A management member agreed that nurse-anaesthetists were not receiving incentives unlike nurses and doctors. Consequently, the hospital supported the nurse-anaesthetists to set up an anaesthetic clinic, where they attended to clients in order to get some extra income. He also agreed that nurse-anaesthetists were a marginalised group and sometimes they were not involved in major decisions that affected them:

When we were renovating the theatre, the nurses went to take the nice rooms for their own restrooms and their storerooms. The doctors already have one room for resting, where will the anaesthetists stay? … Anaesthetists were an afterthought and then they went and positioned them outside the theatre.

Nurse-anaesthetists responded to perceived injustice by using ‘avoiding’ strategy including not pasting their schedule on the notice board, so that no nurse-anaesthetist could be held accountable. They also sometimes left work before scheduled times, and other times they skipped work. Resultantly, sometimes elective C/Ss were delayed, because when other parties were ready, the scheduled nurse-anaesthetist was absent or late. Delays contributed to some elective C/Ss cases progressing into emergency cases. This resulted in heavy workload and pressure on workers in the theatre and wards, which contributed to tensions and petty quarrels that poisoned the organisational climate.

Over time frequent conflicts became the norm, so workers who experienced conflicts did not report such incidences to department and hospital management for redress.

## Discussion

This article contributes to knowledge on the influence of conflicts on health workers’ motivation in the provision of maternal and neonatal health care. Organisational gaps including unclear lines of authority between doctors and nurse-anaesthetists, perceived distributive and interactional injustice, organisational arrangements and communication gaps and a lack of team spirit contributed to frequent conflicts. Individual factors such as doctors and nurse-anaesthetists’ different professional orientation and personal differences contributed to conflicts in decisions on client care. Interpersonal factors including distrust between doctors and nurse-anaesthetists based on locum practice, distrust of professional competencies and perception of who has the ultimate decision making power also contributed to conflicts. Lack of effective conflict management mechanisms intensified conflicts situations, which frustrated workers and affected their motivation to collaborate in the provision of health care. However conflicts that resulted from workers’ determination to ensure that quality health care was provided to clients motivated such workers.

The study found that conflicts in the operating theatre were more frequent between doctors and nurse-anaesthetists. This finding contrasts with studies that suggest that conflicts in the operating theatre are more common between doctors and theatre nurses [46,47]. Nevertheless disagreements over roles, schedules and goals, which were noted as the causes of the conflicts between doctors and theatre nurses [47], were also some of the causes of the conflicts between doctors and nurse-anaesthetists.

Doctors and nurse-anaesthetists disagreed on who had the ultimate authority to decide on client care, which contributed to frequent conflicts. Similarly Fox [48] notes that the doctor has the power to admit the client into the ward and plan the surgery. However in the operating theatre the anaesthetist becomes equally powerful, because the doctor is dependent on his input for a successful surgery. So when the two are not able to come to a consensus conflicts occur.

Doctors favoured intervening promptly in client care to remove or reduce the disease, while nurse-anaesthetists favoured a holistic understanding of a client’s condition before deciding whether surgery should be performed. This different decision pathways contributed to conflicts. Similarly, Fox's [48] study in selected British hospitals notes that the surgical discourse perceives the client as carrier of disease or illness, which should be removed or reduced, while the anaesthetic discourse sees the client as possessor of a complement of fitness, which the anaesthetist should maintain. He suggests that this different orientation influence each professional groups decision making pathways, which contributes to frequent disagreements and conflicts between doctors and anaesthetists.

Organisational arrangements and poor communication contributed to poor team spirit and distrust relations that contributed to conflicts between doctors and nurse-anaesthetists in the hospital. Similarly, Pavlakis et al. [49] and Brinkert [22] suggest that poor communication is a major cause of co-worker conflicts, which can lead to delays in carrying out procedures. Conflicts resulting from communication gaps and weak team spirit between doctors and anaesthetists in the operating theatre have also been documented in the UK [50] and the Canadian [51] health care systems.

Nurse-anaesthetists’ perception of distributive and interactional injustice at organisational and interpersonal levels contributed to conflicts with doctors. Booij [46] study corroborates this finding that anaesthetists’ perception of unfair treatment from the organisation and doctors is common. He attributes this to hospital politics, which perceives anaesthetists as an expense and not an asset to the hospital and also anaesthetists’ feel unappreciated by surgeons.

Distrust relations resulting from locum practice and illegal charges contributed to the occurrence of conflicts, which was a source of demotivation to both doctors and nurse-anaesthetists. Similarly, Bousari et al.'s [52] study found that health workers having other jobs affected work schedule, which frustrated collaborative efforts of their colleagues and contributed to conflicts.

Doctors and nurse-anaesthetists who prevented co-workers who they deemed not competent to perform particular tasks were motivated in situations that their actions created conflicts, but it resulted in clients receiving care from competent professionals. This suggests that conflicts are not always negative, they can help to prevent medical complacency and negligence, which is essential for quality health care provision. Our finding supports studies that suggest that conflicts are not necessarily negative, but can enhance workers’ motivation and performance and contribute to organisational growth and development [2,21,23,24,53]. For instance Smyth’s study found that conflicts increased creativity and the development of innovation and also increased workers’ motivation [24].

Health sector employment in Ghana is centralised and beyond the control of hospital managers. Resultantly department and hospital mangers had no power to hire and fire permanent workers. They could only give verbal and written queries to doctors and nurse-anaesthetists, who used part of their official work hours to practice locum and those who charged illegal fees. This national arrangement as well as the specialised nature of their skill, gave doctors and nurse-anaesthetist a lot of power, which they used to influence organisational processes and practices in Adom hospital. As noted by Lipsky [40] and Mintzberg [39], their decisions and actions in implementation effectively became the policies of the hospital.

Most conflicts were not reported to the department and hospital management, because there was no clear organisational structure for reporting and managing conflicts. Consequently conflicts persisted and became part of the organisational culture. Similarly Katz's [51] study notes that poor conflict management destroys professional relationships and team work, which is essential for client care. Other studies suggest that conflicts are common in health care organisations in most parts of the world including the USA [54] and the UK [47]. Rosenstein [54] suggests that they continue to exist, because they are tolerated and most doctors do not see them as conflicts, but as part of the game of protecting the client’s interest.

Frequent conflicts had multiple effects on frontline workers, the hospital, as well as indirect and direct effects on client quality care. Conflicts contributed to health workers feeling frustrated, experiencing lack of job satisfaction and lack of personal joy in working. At the hospital level there was poor organisational climate and workers lacked the desire to collaborate. This resulted in indirect effects on quality health care including workers feeling demotivated and exhibiting negative attitudes towards clients, which hampered the hospital’s goal of providing quality care. The direct effects included delays in health care provision and sometimes failure to provide essential care to clients. The negative effects of conflicts on health workers’ wellbeing have been reported [54–56]. At hospital level various studies confirm that conflicts can lead to poor organisational climate, low productivity and worker turn over [46,55,56]. A study of mental health workers in Ghana found that the reverse is true. Good work relationship with colleagues is a motivator to workers [35]. Other studies suggest that the direct effects of conflicts on clients’ quality care include compromising client safety, possible medical errors and sometimes mortality among clients [46,55,56]. Indirect effects include a poor responsive health care system to client health care needs, thus decreasing their satisfaction [46,55,56].

## Conclusion and Policy Implications

In a poor resource setting like Ghana, where judicious use of resources is of essence, frequent co-worker conflicts deny hospitals optimum use of scarce human resources. Frequent conflicts result in clients receiving poor quality care not only because of the absence of skilled human resources, but also from poor management. Consequently, understanding the causes of conflicts and how to manage them is crucial for the wellbeing of workers, the organisation and clients.

The hospital needs to see frequent conflicts as a threat to quality health care provision. As a preventive measure, the causes of conflicts need to be investigated in order to address them holistically. This will require that hospital managers, departmental managers and frontline workers engage in regular and constant dialogue to discuss potential causes of conflicts and make broad consultations on how to address them and also commit themselves to addressing them.

Resources should be committed to frequent training workshops for managers and frontline workers to improve their skills in identifying and managing conflicts. This will enable workers to reduce their frequency and severity. A reduction in the frequency of conflicts can foster positive relations between doctors and nurse-anaesthetists, which can help improve collaboration in health care provision.

Organisational guidelines for reporting conflicts and unethical professional conduct and professional incompetence should be put in place to enable workers to report such practices to the appropriate authority. Also measures should be established to correct such practices in the hospital.

Structures should be put in place at ward, department and hospital levels to manage conflicts between individual health workers, as well as between professional groups, by resorting to collaborative response, through addressing the concerns of all conflicting parties. The formation of an anaesthetist clinic is a step towards addressing incentive gaps. However, hospital management can go a step further to address the perception of injustice by involving representatives of the various professional groups in decision making at organizational level, especially when such matters have a direct bearing on them.

Hospital managers should aim at promoting strong team spirit between the different professionals, since maternal and neonatal health care requires team work. This will require that managers imbibe in all professionals the need to respect and appreciate each other and to communicate more effectively on client care. Regular meetings and interactions between doctors, nurse-anaesthetists, nurses and hospital managers to identify, discuss and address challenges of the different professional groups and the hospital should be promoted. This can help workers to understand one another and develop a common sense of purpose, which is essential for the building of team spirit. In sum a good team spirit will foster collective response to client care, which will facilitate the provision of quality care to clients. Hopefully, this will contribute to the effective use of human resources towards the achievement of MDGs 4 and 5.

## References

[1] Franco LM, Bennett S, Kanfer R (2002) Health sector reform and public sector health worker motivation: a conceptual framework. Social Science and Medicine 54: 1255–1266. 11989961 10.1016/s0277-9536(01)00094-6

[2] Almost J (2006) Conflict within nursing work environments: concept analysis. Journal of Advanced Nursing 53: 444–453. 16448487 10.1111/j.1365-2648.2006.03738.x

[3] Coombs M (2003) Power and conflict in intensive care clinical decision making. Intensive and Critical Care Nursing 19: 125–135. 12765632 10.1016/s0964-3397(03)00040-5

[4] Gilson L (2003) Trust and the development of health care as a social institution Social Science and Medicine 56: 1453–1468. 12614697 10.1016/s0277-9536(02)00142-9

[5] Rowe AK, de Savigny D, Lanata CF, Victora CG (2005) How can we achieve and maintain high-quality performance of health workers in low-resource settings? Lancet 366: 1026–1035. 16168785 10.1016/S0140-6736(05)67028-6

[6] Dieleman M, Cuong PV, Anh LV, Martineau T (2003) Identifying factors for job motivation of rural health workers in North Viet Nam. Human Resources for Health 1: 1–10. 14613527 10.1186/1478-4491-1-10 PMC280735

[7] Dieleman M, Toonen J, Touré H, Martineau T (2006) The match between motivation and performance management of health sector workers in Mali. Human Resources for Health 4: 1–7. 16469107 10.1186/1478-4491-4-2 PMC1402315

[8] Purohit B, Bandyopadhyay T (2014) Beyond job security and money: driving factors of motivation for government doctors in India. Human Resources for Health 12. 10.1186/1478-4491-12-12 PMC3936698 24555787

[9] Franco L M, Bennett S, Kanfer R, P. S (2004) Determinants and consequences of health worker motivation in hospitals in Jordan and Georgia. Social Science and Medicine 58: 343–355. 14604620 10.1016/s0277-9536(03)00203-x

[10] Agyepong I, Anafi P, Ansah E, Ashon D, Na-Dometey C (2004) Health Worker (internal customer) satisfaction and motivation in the public sector in Ghana. International Journal of health planning and Management 19: 319–336. 15688876 10.1002/hpm.770

[11] Ansong-Tornui J, Armar-Klemesu M, Arhinful D, Penfold S, Hussein J (2007) Hospital based maternity care in ghana—findings of a confidential enquiry into maternal deaths. Ghana Med J 41: 125–132. 18470330 10.4314/gmj.v41i3.55280 PMC2279086

[12] Bosu W, Bell J, Armar-Klemesu M, Ansong-Tornui J (2007) Effect of Delivery Care User Fee Exemption Policy on Institutional Maternal Deaths in the Central and Volta Regions of Ghana. Ghana Med J 41: 118–124. 18470329 10.4314/gmj.v41i3.55278 PMC2279091

[13] National Development Planning Commission, Government of Ghana, United Nations Development Programme (UNDP) Ghana (2010) 2008 Ghana Millennium Development Goals report. Accra: National Development Planning Commission (NDPC), Government of Ghana and the United Nations Development Programme (UNDP) Ghana.

[14] Bonenberger M, Aikins M, Akweongo P, Wyss K (2014) The effects of health worker motivation and job satisfaction on turnover intention in Ghana: a cross-sectional study. Human Resources for Health 12: 1–12. 25106497 10.1186/1478-4491-12-43 PMC4130118

[15] Agyepong I, Sollecito W, Adjei S, Veney J (2001) Continues quality improvement in public health in Ghana: CQI as a model for primary health care management and delivery. Quality Management in Health Care 9: 1–10. 10.1097/00019514-200109040-00002 11499347

[16] Mathauer I, Imhoff I (2006) Health worker motivation in Africa: the role of non-financial incentives and human resource management tools. Human resources for Health 4: 1–17. 16939644 10.1186/1478-4491-4-24 PMC1592506

[17] Brinkert R (2010) A literature review of conflict communication causes, costs, benefits and interventions in nursing. Journal of Nursing Management 18: 145–156. 10.1111/j.1365-2834.2010.01061.x 20465742

[18] Bercovitch J (1983) Conflict and conflict management in Organisations: A framework for analysis. The Asian Journal of Public Administration 5: 104–123.

[19] Almost J, Doran DM, Hall LM, Laschinger HKS (2010) Antecedents and consequences of intra-group conflict among nurses. Journal of Nursing Management 18: 981–992. 10.1111/j.1365-2834.2010.01154.x 21073570

[20] Cinar F, Kaban A (2012) Conflict Management and Visionary Leadership: An Application in Hospital Organizations. Social and Behavioral Sciences 58: 197–206.

[21] Slack T, Parent MM (2006) Understanding sport Organisations: The application of organisation theory United States of America: Sheridan Books.

[22] Whetten DA, Cameron K, Woods M (1996) Effective conflict management; Whetten DA, Cameron KS, editors. Hammersmith, London: HarperCollins Publishers.

[23] Pondy LR (1967) Organizational Conflict: Concepts and Models. Administrative Science Quarterly 12: 296–320.

[24] Vivar CG (2006) Putting conflict management into practice: a nursing case study. Journal of Nursing Management 14: 201–2006. 16600008 10.1111/j.1365-2934.2006.00554.x

[25] Zydziunaite V, Katiliute E (2007) Improving motivation among health care workers in private health care organizations: A perspective of nursing personnel. Baltic Journal of Management 2: 213–224.

[26] De Dreu CKW, Weingart LR (2003) Task Versus Relationship Conflict, Team Performance and Team Member Satisfaction: A Meta-Analysis. Journal of Applied Psychology 88: 741–749. 12940412 10.1037/0021-9010.88.4.741

[27] Jehn KA, Rupert J, Nauta A (2006) "The effects of conflict asymmetry on mediation outcomes". International Journal of Conflict Management 17: 96–109.

[28] Laschinger HKS, Finegan J, Shamian J, Casier S (2000) Organizational Trust and Empowerment in Restructured Healthcare Settings: Effects on Staff Nurse Commitment. The Journal of Nursing Administration 30: 413–425. 11006783 10.1097/00005110-200009000-00008

[29] Thom D, Hall M, Pawlson G (2004) Measuring patients’ trust in Physicians when assessing quality of care. Health Affairs 23: 124–132. 10.1377/hlthaff.23.4.124 15318572

[30] Gilson L, Palmer N, Schneidera H (2005) Trust and health worker performance: exploring a conceptual framework using South African evidence. Social Science & Medicine 61: 1418–1429. 16005777 10.1016/j.socscimed.2004.11.062

[31] Gilson L (2006) Trust in health care: theoretical perspectives and research needs. Journal of Health Organization and Management 20 359–375. 17087400 10.1108/14777260610701768

[32] Ferres N, Connell J, Travaglione A (2004) Co-worker trust as a social catalyst for constructive employee attitudes. Journal of Managerial Psychology 19: 608–622.

[33] Johns JL (1996) A concept analysis of trust. Joumal of Advanced Nursing 24: 76–63. 10.1046/j.1365-2648.1996.16310.x 8807380

[34] Gilson L, Palmer N, Schneider H (2005) Trust and health worker performance: exploring a conceptual framework using South African evidence. Social Science & Medicine 61: 1418–1429. 16005777 10.1016/j.socscimed.2004.11.062

[35] Jack H, Canavan M, Ofori-Atta A, Taylor L, Bradley E (2013) Recruitment and Retention of Mental Health Workers in Ghana. PLoS ONE 8: 1–8. 10.1371/journal.pone.0057940 PMC3585225 23469111

[36] Zand DE (1972) Trust and Managerial Problem Solving. Administrative Science Quarterly 17: 229–239.

[37] Fox A (1974) Beyond Contract: Work, Power and Trust Relations London: Faber & Faber

[38] Mansour JB, Vriesendorp S, Ellis A (2005) Managers who lead: A handbook for improving Health Services; Miller J, Bahamon C, Timmons BK, editors. USA: Quebecor World.

[39] Mintzberg H (1983) Power in and around organisations USA: Prentice-Hall.

[40] Lipsky M (1980) Street-level Bureaucracy:Dilemmas of the individual in Public services New York: Russell Sage Foundation.

[41] Dahl RA (1957) The Concept of Power Journal of Behavioral Science 2: 201–215.

[42] Van der Geest S, Sarkodie S (1998) The fake patient: A research experiment in a Ghanaian Hospital. Soc Sci Med 47: 1373–1381. 9783880 10.1016/s0277-9536(98)00179-8

[43] Emerson RM, Fretz RI, Shaw LL (2005) Writing ethnographic fieldnote Chicago: The University of Chicago Press pp. 353–368.

[44] Discombe G (1954) The normal blood count. British Medical Journal 1: 326–328. 13115715 10.1136/bmj.1.4857.326 PMC2093322

[45] Encyclopedia Wikipedia (2014) Uterine myomectomy Wikipedia the Free Encyclopedia: Wikimedia Foundation, Inc.,.

[46] Booij LHDJ (2007) Conflicts in the operating theatre. Current Opinion in Anaesthesiology 20: 152–156. 17413400 10.1097/ACO.0b013e32809f9506

[47] Coe R, Gould D (2007) Disagreement and aggression in the operating theatre. Journal of Advanced Nursing 61: 609–618. 10.1111/j.1365-2648.2007.04544.x 18302602

[48] Fox NJ (1994) Anaesthetists, the discuorse on patient fitness and the organisation of surgery In: Board E, editor. Basil Blackwell limited. Oxford, UK: Blackwell Publishers.

[49] Pavlakis A, Kaitelidou D, Theodorou M, Galanis P, Sourtzi P, Siskou O (2011) Conflict management in public hospitals: the Cyprus case. International Nursing Review 58: 242–248. 10.1111/j.1466-7657.2011.00880.x 21554299

[50] Mohammed A, Wu J, Biggs T, Duffy S (2012) Differences in the perception of urgency of cesarean delivery between obstetricians and anesthetists. International Federation of Gynecology and Obstetrics: Brief Communications. 10.1016/j.ijgo.2012.02.002 22424661

[51] Katz JD (2007) Conflict and its resolution in the operating room. Journal of Clinical Anesthesia 19: 152–158. 17379132 10.1016/j.jclinane.2006.07.007

[52] Bousari PM, Ebrahimi H, Ahmadi F, Abedi HA, Kennedy N (2009) The Process of Nurses Interpersonal Conflict: Qualitative Study. Research Journal of Biological Sciences 4: 236–243.

[53] Guerra ST, Prochnow AG, Trevizan MA, Guido LdA (2011) Conflict in Nursing Management in the Hospital Context. Rev Latino-Am Enfermagem 19: 362–369. 10.1590/s0104-11692011000200019 21584384

[54] Rosenstein AH (2011) Managing disruptive behaviors in the health care setting: focus on obstetrics services. American Journal of Obstetrics & Gynecology: 187–192. 10.1016/j.ajog.2010.10.899 21183152

[55] Capitulo KL (2009) Addressing Disruptive Behavior by Implementing a Code of Professionalism to Transform Hospital Culture. Nurse Leader 7: 38–43.

[56] Bazin J-E, Attias A, Baghdadi H, Baumann A, Bizouarn P, Claudot F et al (2014) Perioperative conflicts between anaesthesiologists and surgeons: Ethics and professionalism. Annales Franc¸aises d’Anesthe´ sie et de Re´animation 33: 335–343. 10.1016/j.annfar.2014.04.006 24821342

